# Teaching and learning styles in quality improvement: Identification and impact on process outcomes

**DOI:** 10.1186/1940-0640-10-S1-A12

**Published:** 2015-02-20

**Authors:** James H Ford, James Robinson

**Affiliations:** 1 Center for Health Systems Research and Analysis, University of Wisconsin-Madison, Madison, WI, 53726, USA

## Background

A quality improvement collaborative uses multiple approaches, including coaching, to teach the necessary skills to the change leader in an effort to teach them quality improvement skills and to enhance their knowledge acquisition in order to implement effective organizational changes. However, little is known about the individual teaching and learning styles of the coach or change leader and how a particular style or a match between teaching and learning style influence quality improvement outcomes. This study builds on the NIATx200 results. It seeks to answer two research questions: 1) What are the learning and teaching style typology in a quality improvement collaborative? (2) How do levels of convergence and divergence between staff learning style and coach teaching style influence the outcomes in a quality improvement collaborative?

## Methods

The Grasha-Riechmann Student Learning Style survey and Teaching Style Inventory, developed and validated within the field of educational research, were modified to identify the individual teaching and learning styles of participants in a quality improvement collaborative. Change leaders, executive sponsor, and coaches were invited to complete the surveys. Using NIATx200 results, each outcome (wait time, continuation, and admissions) was classified as improved or not improved for each site. A pooled factor effect backwards stepwise elimination regression model explored the relationship between the different styles and the NIATx200 outcomes.

## Results

Coaches (n = 17) in a QIC exhibit similar teaching styles identified in an educational setting. The learning style of change leaders (n = 77) in a quality improvement collaborative differs from how students learn in an education setting. Results indicate the presence of 10 different learning styles. The regression results indicate that higher competitive leader learning styles scores is associated with lower wait-time improvements (F = 2.26; p = 0.075) for providers in the learning session intervention. Higher expert and personal model teaching styles are associated with wait time improvements for the coaching and combination arm (F = 3.13; p = 0.01). Coaches with higher expert teaching style scores showed greater admission improvements in the coaching intervention arm (F = 2.42; p = 0.052).

## Conclusions

The preliminary results suggest that certain individual learning and teaching styles influence organizational outcomes for certain interventions within a quality improvement collaborative. Further research is required to understand how teaching and learning styles interact to influence outcome improvement. The findings could suggest how to tailor a quality improvement collaborative to improve outcomes.

